# Tris[*N*-(prop-2-en-1-yl)hydrazinecarbothioamide]-Cobalt(III) Chloride as a Stimulator of the Total Antioxidant Status of *Apis mellifera* with Acaricidal Activity

**DOI:** 10.3390/antiox15010018

**Published:** 2025-12-22

**Authors:** Olga Garbuz, Valeriu Fala, Gheorghe Bordeniuc, Vasilii Graur, Jenny Roy, Nadejda Railean, Victor Tsapkov, Veronica Sardari, Ion Toderas, Aurelian Gulea

**Affiliations:** 1 Laboratory of Systematics and Molecular Phylogenetics, Institute of Zoology, Moldova State University, 1 Academiei Street, MD-2028 Chisinau, Moldova; 2 Laboratory of Advanced Materials in Biopharmaceutics and Technics, Institute of Chemistry, Moldova State University, 60 Mateevici Street, MD-2009 Chisinau, Moldova; 3 Department of Dentistry, State University of Medicine and Pharmacy “Nicolae Testemitanu”, 165 Stefan cel Mare si Sfant Bd., MD-2004 Chisinau, Moldova; 4 Laboratory of Medicinal Chemistry, CHU de Québec Research Center, Université Laval, Québec, QC G1V 4G2, Canada; 5 Department of Biochemistry and Clinical Biochemistry, State University of Medicine and Pharmacy “Nicolae Testemitanu”, MD-2004 Chisinau, Moldova

**Keywords:** tris[*N*-(prop-2-en-1-yl)hydrazinecarbothioamide]-cobalt(III) chloride, antioxidant activity, *Apis mellifera*, total antioxidant status, *Varroa destructor*

## Abstract

This study investigates the synthesis and potential applications of the coordination compound cobalt(III) complex tris[*N*-(prop-2-en-1-yl)hydrazinecarbothioamide]-cobalt(III) chloride (**[Co(Tsc)_3_]Cl_3_**). The complex has been synthesized via the reaction of cobalt(II) chloride hexahydrate with *N*-(prop-2-en-1-yl)hydrazinecarbothioamide in ethanol. Its antioxidant activity has been evaluated using 2,2′-azinobis-(3-ethylbenzothiazoline-6-sulfonic acid (ABTS) assay, demonstrating a significant effect with an IC_50_ of 7.3 µmol/L. Toxicity evaluations using *Daphnia magna* showed a low half maximal inhibitory concentration (LC_50_) of 56.3 µmol/L. Experimental results have showed that **[Co(Tsc)_3_]Cl_3_** significantly elevated the total antioxidant status (TAS) of the hemolymph of honeybees and larvae, increasing it by 5 and 8 times, respectively. The IC_50_ values for antioxidant activity were 2.5 mg/mL in bee hemolymph and 1.3 mg/mL in larval hemolymph, notably lower than control values of 13.6 mg/mL and 10.0 mg/mL. The stimulatory effect of the coordination compound **[Co(Tsc)_3_]Cl_3_** on TAS was five times higher than that of vitamin C. Additionally, **[Co(Tsc)_3_]Cl_3_** exhibited acaricidal properties, effectively inhibiting *Varroa destructor* with an lethal concentration (LC_50_) of 0.2 µmol/L. These findings indicate that this cobalt complex could serve both a natural antioxidant and an effective acaricide, offering a promising approach to improv bee health and sustainability in apiculture.

## 1. Introduction

European honey bees (*Apis mellifera*) play a crucial ecological and economic role through their pollination of both natural ecosystems and agricultural crops, as well as their importance in beekeeping [1,2,3,4,5].

The health and productivity of *Apis mellifera* honeybee colonies critically depend on an efficient antioxidant system that neutralizes active free radicals generated during the high metabolic activity [6,7,8,9]. Insects such as bees naturally produce a significant amount of free radicals, making them particularly vulnerable to oxidative stress [10]. Oxidative stress not only disrupts cellular functions but also considerably contributes to the development of various diseases, ultimately impacting the vitality and survival of the colonies [11,12].

A major factor exacerbating the oxidative stress in bees is infestation by the parasitic mite *Varroa destructor*. This mite feeds on hemolymph and damages vital tissues, thereby increasing the production of active free radicals and overwhelming the antioxidant defenses. This accelerates aging processes, weakens the immune system, and ultimately leads to colony collapse [7,13,14,15,16,17].

The assessment of antioxidant activity (AOA) serves as a critical indicator of the functional state of the antioxidant defense system in bees and their larvae [18,19,20,21,22,23,24,25,26,27,28,29]. A key parameter in this regard is the total antioxidant status (TAS) of *Apis mellifera*, which reflects the cumulative activity of both enzymatic and non-enzymatic antioxidants in the organism [30,31].

Although vitamin C is widely used to enhance the TAS of *Apis mellifera*, its relatively low antioxidant capacity limits its effectiveness [25,26,32,33]. Recent studies have explored alternative compounds [34,35,36,37], including transition metal complexes, due to their potential to more effectively enhance antioxidant defenses [38,39,40,41,42,43,44]. Among these, coordination compounds offer promising potential due to their unique chemical properties; however, their application in apiculture remains largely unexplored. Coordination compounds are chemical complexes consisting of a central metal atom or ion bonded to one or more ligands, which are molecules or ions that donate electron pairs to the metal, forming stable chemical bonds. Notably, research on the use of metal coordination complexes for bee health has yielded contradictory results: some hypotheses suggest significant protective effects, while others indicate potential toxicity or limited efficacy [45]. These contradictions underscore the necessity for rigorous exploration of new compounds that possess both antioxidant properties and non-toxicity, ensuring their safety and efficacy in improving bee health and resilience [46,47].

In our studies, we have examined the antioxidant activity of bee hemolymph and larvae, as well as have evaluated the effect of the tested substance on the level of antioxidant protection in bees and larvae of *Apis mellifera*. The tested coordination compound acts as an antioxidant in the bees’ organism, reducing the level of free radicals. The findings of our studies show that supplementation of honey bee nutrition with the coordination compound **[Co(Tsc)_3_]Cl_3_** increases the efficiency of their antioxidative system. Thus, the detected properties of the coordination compound **[Co(Tsc)_3_]Cl_3_** are of interest to biotechnology and beekeeping, expanding the arsenal of stimulators of the total antioxidant status of *Apis mellifera* hemolymph [48,49].

Additionally, we have found that the coordination compound **[Co(Tsc)_3_]Cl_3_** possesses acaricidal property and effectively inhibits *Varroa destructor.* Our data highlight the importance of developing new substances capable not only of enhancing antioxidant defenses but also of acting as acaricide, thus possessing current requirements for protecting bees from parasitic infections and environmental stressors.

## 2. Materials and Methods

### 2.1. Materials

All the used reagents were chemically pure. Cobalt(II) chloride hexahydrate (Merck, Darmstadt, Germany) was used as supplied. Allyl isothiocyanate, 50–60% (*w*/*w*) aqueous solution of hydrazine, and hydrochloric acid were used as received (Sigma-Aldrich, Darmstadt, Germany). The ^1^H and ^13^C NMR spectra were recorded on a Bruker DRX-400 (Ettlingen, Germany) using acetone-d_6_ as a solvent. FT-IR spectra were recorded on a Bruker ALPHA FTIR spectrophotometer at room temperature in the range of 4000–400 cm^−1^. UV–Vis spectra were measured on Agilent Cary 300 UV–Vis spectrophotometer (Santa Clara, CA, USA) in EtOH solutions within wavelength 200–100 nm. The elemental analysis was performed similarly to the literature procedures [50], and on the automatic Perkin Elmer 2400 elemental analyzer (Rodgau, Germany). R-38 rheochord bridge was used to measure the resistance of 1 mM methanol solutions of the coordination compound at 20 °C.

4-Allylthiosemicarbazide was prepared by reaction of the allyl isothiocyanate and a 50–60% (*w*/*w*) aqueous solution of hydrazine. The characteristics of the obtained substance correspond with the data reported in the literature [51].

White solid. Yield: 84%; mp 92–93 °C. FW: 131.20 g/mol; Anal Calc. for C_4_H_9_N_3_S: C, 36.62; H, 6.91; N, 32.03; S, 24.44; found: C, 36.48; H, 6.83; N, 32.09; S, 24.51%. ^1^H NMR (acetone-d_6_, 400 MHz) 9.03 (br s, 1H; NH); 7.86 (br s, 1H; NH); 5.92 (m, 1H; CH); 5.13 (m, 2H; CH_2_); 4.39 (br s, 1H; NH); 4.25 (t, 2H; CH_2_–N). ^13^C NMR (acetone-d_6_, 100 MHz) 178.86 (C=S); 134.76 (CH(_allyl)_); 114.85 (CH_2(allyl,sp_^2^_)_); 45.85 (CH_2(allyl,sp_^3^_)_). FTIR data (cm^−1^): ν(NH, –NH_2_) 3338, 3268, 3191; ν (C=N) 1640, 1628; ν (C=S) 1284. λ, nm (ε, M−1 cm^−1^): 206 (13,300), 241 (11,900).

### 2.2. Synthesis of [Co(Tsc)_3_]Cl_3_

Cobalt(II) chloride hexahydrate (CoCl_2_·6H_2_O) (2.38 g, 10 mmol) was added to a hot (55° C) ethanolic solution (25 mL) of 4-allylthiosemicarbazide (Tsc) (3.94 g, 30 mmol). 37% (w/w) aqua solution of HCl (0.83 mL, 10 mmol) was added to the reaction mixture. The resulting mixture was stirred for 1 h at 55 °C under continuous air bubbling. By cooling to room temperature, a brown precipitate was obtained. It was filtered out, washed with cold ethanol, and dried in vacuo.

Brown solid. Yield: 87%. Anal. Calc. for C_12_H_27_Cl_3_CoN_9_S_3_ (558.87 g mol^−1^): C, 25.79; H, 4.87; Cl, 19.03; Co, 10.54; N, 22.56; S, 17.21. Found: C, 25.95; H, 4.78; Cl, 19.32; Co, 10.72; N, 22.83; S, 7.42. Main FTIR peaks (cm^−1^): ν(NH, –NH_2_) 3251, 3199, 3079; ν(C=N) 1618, 1581; ν(C=S) 1285. λ, nm (ε, M^−1^ cm^−1^): 214 (22,900), 261 (21,900), 544 (370). χ(CH_3_OH): 312 Ω^−1^ cm^−2^ mol^−1^.

### 2.3. Enriched Feeding Technique

Biological trials were conducted on colonies of *Apis mellifera* honeybees. For the administration of the coordination compound **[Co(Tsc)_3_]Cl_3_** complex, a syrup (50% sugar, 50% water) was used. The tested substance was administered at a concentration of 0.2 mg/L and 0.6 mg/L in the syrup. The syrup was used to feed the bees at the beginning of spring, when nectar and pollen sources are limited.

Experiments were carried out simultaneously on three groups, each consisting of 10 hives. Group I served as the control, where bees received plain sugar syrup without the tested complex. Groups II and III were used for studying the tested complex, and the bees were fed syrup enriched with coordination compound **[Co(Tsc)_3_]Cl_3_** at concentrations of 0.2 mg/L and 0.6 mg/L, with doses of 2 mg and 6 mg per hive, respectively.

Feeding was carried out by adding syrup into a container placed in the upper part of the hive. The volume was 100 mL of syrup per frame. The syrup was added every two days over a period of two weeks.

### 2.4. ABTS^•+^ Radical Cation Scavenging Assay

The antioxidant activity by the ABTS^•+^ method was assessed according to the method described by Re et al. [52] with modifications. An ABTS^•+^ assay is an excellent tool for determining the antioxidant activity of hydrogen-donating antioxidants and chain-breaking antioxidants.

The ABTS^•+^ radical was formed through the reaction of ABTS (2,2′-azino-bis(3-ethylbenzothiazoline-6-sulphonic acid)) (Sigma) solution 7 mM with potassium persulfate (K_2_S_2_O_8_) (Sigma, Germany) solution, incubated at 25 °C in the dark for 12–20 h at room temperature. The resulting solution was further diluted by mixing with ethanol (0.02 M, pH 6.5) to obtain an absorbance of 0.70 ± 0.01 units at 734 nm.

20 μL of each tested dilutions was transferred to a 96-well microtiter plate and 180 μL of working solution of ABTS^•+^ was dispensed with the dispense module of the hybrid reader (BioTek, Santa Clara, CA, USA). The decrease in absorbance at 734 nm was measured exactly after 30 min of incubation at 25 °C. All the determinations were made in triplicate. Blank samples were run by solvent without ABTS^•+^.

The measurement was made by a hybrid reader (Synergy H1, BioTek). All tests were performed in triplicate and the obtained results were averaged. The percent of inhibition (*I*, %) of free radical cation production of ABTS^•+^ was calculated by using the following equation:

(1) $$I\;(\%)\;=\frac{{{Abs}_{734\;nm}}_{0}\;-\;{{Abs}_{734\;nm}}_{1}}{{{Abs}_{734\;nm}}_{0}}\times 100\;\;\;$$

*Abs*_734 nm0_ is the absorbance of the control solution;

*Abs*_734 nm1_ is the absorbance in the presence of sample solutions or standards for positive controls.

### 2.5. DPPH^•^ Radical Scavenging Assay

The DPPH^•^ assay was done according to the method of Brand-Williams et al. with some modifications. The effect of antioxidants on DPPH^•^ radical scavenging is due to the hydrogen-donating ability or radical scavenging activity of the samples. The scavenging reaction between (DPPH) and an antioxidant (H-D) can be written as: (DPPH) + (H-D) → DPPH-H + (D). The DPPH reagent was diluted with a methanolic solution to give an absorbance of 0.7 ± 0.01 at 517nm.

The tested dilutions 20 μL were transferred in a 96 wells microtitre plate, and 180 μL of DPPH^•^ (1,1-diphenyl-2-picrylhydrazyl) reagent 0.002% *w/v* (Sigma) with ethanol was dispensed with dispense module of the hybrid reader (Synergy H1, BioTek), shake 10 s. The decrease in absorbance at 515 nm was measured exactly after 30 min of incubation at 25 °C (Hybrid reader, Synergy H1, BioTek).

All the determinations were made in triplicate. Blank samples were run by solvent without DPPH^•^ radical. The measurement was made by a hybrid reader (Synergy H1, BioTek). All tests were performed in triplicate and the obtained results were averaged. The percent of inhibition (*I*, %) of free radical production of DPPH^•^ was calculated by using the following equation:

(2) $$I\;(\%)\;=\frac{{{Abs}_{517\;nm}}_{0}\;-\;{{Abs}_{517\;nm}}_{1}}{{{Abs}_{517\;nm}}_{0}}\times 100\;\;\;$$

*Abs*_517 nm 0_ is the absorbance of the control solution;

*Abs*_517 nm 1_ is the absorbance in the presence of sample solutions or standards for positive controls.

### 2.6. Assessment of Varroa destructor Infestation Levels

The bees were fed with a 50% sugar, 50% water syrup containing the coordination compound **[Co(Tsc)_3_]Cl_3_** at concentrations of 0.2 mg/L and 0.6 mg/L. The syrup was used to feed the bees in the early spring period. Experiments were carried out simultaneously on three groups, each consisting of 10 hives. Group I served as the control, where bees received plain sugar syrup without the tested coordination compound. Groups II and III were used for studying the tested coordination compound, and the bees were fed syrup enriched with coordination compound **[Co(Tsc)_3_]Cl_3_** at concentrations of 0.2 mg/L and 0.6 mg/L, with doses of 2 mg and 6 mg per hive, respectively. Syrup was added every two days over a period of two weeks.

**Figure 1:**
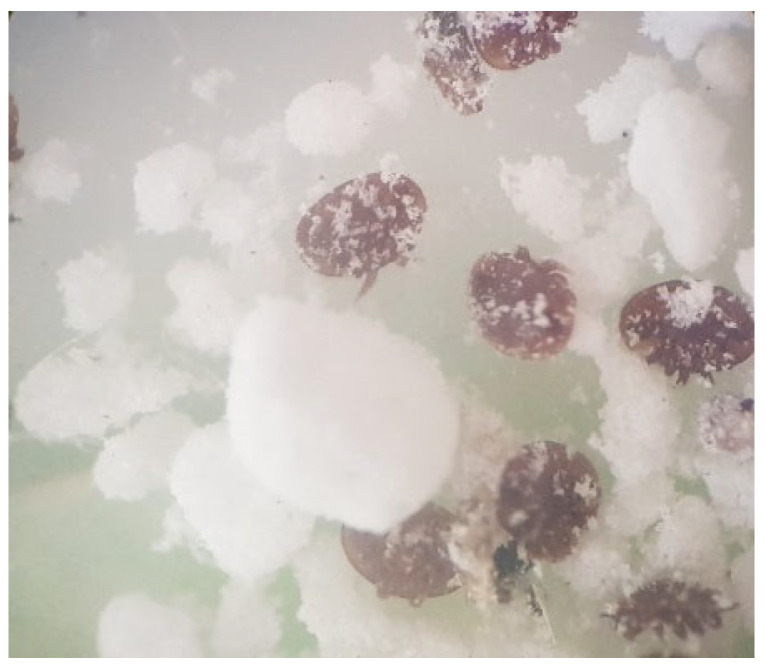
*Varroa destructor* mites in powdered sugar.

To count *Varroa destructor* mites, a non-harmful diagnostic method based on the use of powdered sugar was employed (Figure 1). For the experiment, a 750 mL jar with a perforated lid was used. Bees from a well-developed frame were placed inside the jar. The lid’s holes were then covered, and 10 g of powdered sugar was added to approximately 300 bees inside the jar. After gently shaking the jar for 2 min, the mites fell through the holes in the lid, while the bees remained inside. The bees were then returned to the colony, and the number of mites was counted against a light background. To determine the number of mites per 100 bees, the total mite count was divided by 3, and the result was expressed as a percentage (%). This value reflects the level of *Varroa destructor* infestation in *Apis mellifera* colonies [53].

The degree of brood infestation was assessed by examining 100 larvae of *Apis mellifera* [35].

### 2.7. Statistical Analysis

The antioxidant assay results are presented as the percentage of inhibition (I, %) of DPPH^•^ and ABTS^•+^ radicals. To gauge the efficacy of the experimental compounds on antioxidant activity (AOA), the half-maximal inhibitory concentration values (IC_50_) were computed using the dose–response equation derived from the least squares fit method. All data are expressed as means ± standard deviation (SD).

To accurately assess the effectiveness of the tested substances, Varroa infestation levels were evaluated within each group and compared to the control groups. This approach helps account for potential differences in initial infestation levels across groups and among colonies within groups. Overall, the effectiveness was determined by calculating the percentage (%) reduction in *Varroa destructor* infestation levels in *Apis mellifera* colonies relative to the control groups. For comparative characterization of the acaricidal properties, the median lethal concentration (LC_50_), at which 50% of *Varroa destructor* parasites are killed, has been determined. Statistical analysis has been performed using BIOSTAT (BioStat 2008 5.0.1) and GraphPad (Prism 6) software to process and analyze the obtained data.

## 3. Results and Discussion

**Figure 2:**
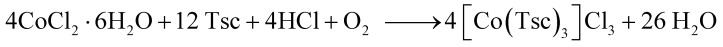
General scheme of the synthesis.

**Figure 3:**
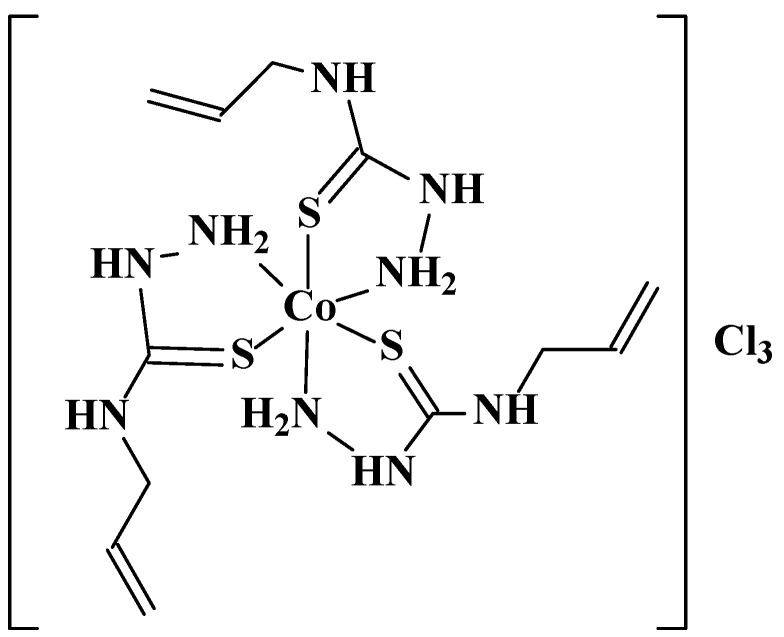
Structural formula of the **[Co(Tsc)_3_]Cl_3_**.

A new cobalt(III) coordination compound (**[Co(Tsc)_3_]Cl**_3_) has been developed to regulate the total antioxidant status (TAS) in the hemolymph of *Apis mellifera.* A cobalt(III) coordination compound has been obtained by interaction of ethanol solution of 4-allylthiosemicarbazide (Tsc), cobalt(II) chloride hexahydrate and hydrochloric acid in aerated medium in a 3:1:1 molar ratio. The general scheme is presented by the following reaction (Figure 2):

The FTIR spectrum of the complex [Co(TSC)_3_]Cl_3_ has been examined and compared with that of the 4-allylthiosemicarbazide (TSC) to evaluate the spectral modifications arising from ligand coordination to the cobalt atom. The analysis indicates that, in the synthesized cobalt(III) complex, the ligand binds to the metal ion through the NH_2_ group and the sulfur atom. The presence and positions of the characteristic absorption bands confirm that the TSC ligand remains in its thione form and does not undergo deprotonation upon coordination. Composition and structure of the coordination compounds has been determined using elemental analysis and molar conductivity measurements. Molar conductivity value (312 Ω cm^2^ mol^−1^, CH_3_OH, 20 °C, 0.001 mol L^−1^) indicates that it represents an electrolyte of an 1:3 type. Its structural formula is presented in Figure 3.

### 3.1. Stimulation of the Total Antioxidant Status of the Hemolymph of Apis mellifera

Metabolic processes in aerobic organisms are a constant source of active free radicals, which serve a dual role: as signaling molecules regulating cellular and intercellular processes and as agents of potential oxidative stress. To maintain cellular homeostasis, organisms must balance the production of free radicals with antioxidant defenses. This balance is critically important, as oxidative stress resulting from an excess of free radicals is a key factor in the initiation and progression of numerous diseases and pathological conditions in bees [54].

Oxidative stress occurs when free radical processes are activated, leading to the peroxidation of lipids, disruption of nucleic acid structures, modification of amino acids in proteins, and subsequent changes in their biological activity [51]. These cellular disruptions ultimately result in tissue damage and cell death, affecting vital organs and tissues in bees, including their larvae.

For bees, especially those with high metabolic rates, like bees, an efficient antioxidant system is vital [55]. Such systems help in neutralizing the excessive free radicals produced as by-products of their metabolism, preventing potential cellular damage. The total antioxidant status (TAS) of an organism is a key indicator used to evaluate the efficiency and functional state of the antioxidant system.

By assessing TAS in bees and their larvae, researchers can gain insights into their ability to cope with oxidative stress, which is integral to maintaining the health and longevity of bee colonies. This understanding is particularly important given the environmental and biological challenges these essential pollinators face.

**Figure 4:**
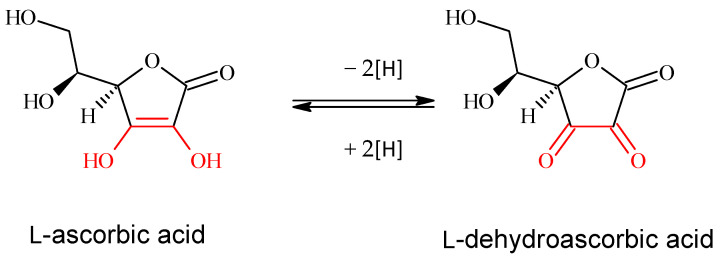
The redox reaction of vitamin C, molecular forms in equilibrium. L-dehydroascorbic acid also possesses biological activity and it is reduced to form ascorbic acid in the organism due to this quality.

Diet is a vital factor in the survival and health of *Apis mellifera* colonies during spring. Implementing strategies to prevent or mitigate the effects of oxidative stress is particularly important for maintaining the organism’s protective defenses. One widely adopted approach involves introducing exogenous compounds with antioxidant properties, among which vitamin C (also known as ascorbic acid) is currently the most common and serves as a prototype. It is extensively used as a standard in biochemical analyses to evaluate the antioxidant activity of both natural and synthetic substances. Within the organism, vitamin C functions in aqueous environments and can be oxidized to dehydroascorbic acid in the presence of metal ions. This oxidized form is then transported into cells via glucose transporters, as illustrated in Figure 4. Vitamin C primarily interacts with vitamin E and carotenoids, working synergistically with antioxidant enzymes to neutralize free radicals. In membrane lipoproteins, vitamin C interacts with vitamin E, regenerating α-tocopherol from its radical form. As a strong reducing agent, vitamin C effectively neutralizes reactive oxygen species (ROS) such as hydrogen peroxide [15,25,26].

Building upon this foundation, a novel cobalt(III) coordination complex with thiosemicarbazone, namely tris[*N*-(prop-2-en-1-yl)hydrazinecarbothioamide]cobalt(III) chloride **([Co(Tsc)_3_]Cl_3_)**, was synthesized. The aim was to evaluate its potential to augment the total antioxidant capacity of *Apis mellifera* hemolymph. The synthesis has been carried out at the Laboratory of Advanced Materials in Biofarmaceutics and Technics, Moldova State University. Sample collection has involved storing hemolymph samples from honeybees and their larvae in sterile, food-grade containers (plastic or glass), followed by preservation and transport under cryogenic conditions using portable freezers to prevent degradation. The biological specimens have been dissolved in 96% ethanol and incubated at 25 °C for a period of 48 h in a controlled thermostat. The experimental assays have been conducted across a concentration range of 0.01 to 100.00 mg/mL to assess dose-dependent effects.

In initial experiments, the antioxidant activity of the complex **[Co(Tsc)_3_]Cl_3_** was evaluated using the ABTS^·+^ assay. The results demonstrated that the IC_50_ value for **[Co(Tsc)_3_]Cl_3_** is 7.3 ± 0.3 µmol/L, whereas for the standard Trolox it is 33.3 ± 0.3 µmol/L, and for vitamin C it is 14.0 ± 0.3 µmol/L (Table 1). Comparative analysis has revealed that **[Co(Tsc)_3_]Cl_3_** exhibits an antioxidant activity approximately five times higher than Trolox and twice that of vitamin C. The significantly lower IC_50_ value indicates a high potency of **[Co(Tsc)_3_]Cl_3_** as an antioxidant, exceeding the standards Trolox and vitamin C, which are commonly used as benchmarks for evaluating antioxidant properties in biochemical studies and for enhancing antioxidant defenses in biological systems. Considering the crucial role of vitamin C in enhancing the overall antioxidant status of bees [25,26], the high antioxidant activity of **[Co(Tsc)_3_]Cl_3_** underscores its potential in supporting bee health and immunity, as well as in reducing oxidative stress levels.

The toxicity of the coordination compound **[Co(Tsc)_3_]Cl_3_** complex was initially assessed using *Daphnia magna* following the ISO 6341:2012 standard [56]. The results have showed that **[Co(Tsc)_3_]Cl_3_** had a relatively low toxicity, with an LC_50_ of 56.3 ± 3.1 µmol/L, indicating a high level of safety. This suggests that **[Co(Tsc)_3_]Cl_3_** can be safely used as a stimulator of bee development, offering an effective and non-toxic option for enhancing bee health.

The experiments assessing the TAS of the hemolymph of worker bees and their larvae have been conducted simultaneously on three groups, each consisting of 10 hives. Group I served as the control, with bees fed with plain sugar syrup without the tested compound. Group II has been designated for the study of the coordination compound **[Co(Tsc)_3_]Cl_3_**, with bees fed with syrup enriched with coordination compound **[Co(Tsc)_3_]Cl_3_** at a concentration of 0.6 mg/L.

The results of the total antioxidant status of the hemolymph of worker bees and their larvae in the control group I (without influents of the coordination compound **[Co(Tsc)_3_]Cl_3_)** are presented in Table 2.

The IC_50_ values obtained from the hemolymph of control worker bees indicate inherently strong antioxidant activity. Specifically, the ABTS^·^ assay has shown IC_50_ values of 13.56 ± 0.37 μg/mL for the hemolymph of adult bees and 9.95 ± 0.70 μg/mL for larvae. In the DPPH^•^ assay, the IC_50_ values were 13.85 ± 0.19 mg/mL for adult bee hemolymph and 13.44 ± 2.95 mg/mL for larvae.

The TAS of hemolymph of bees and their larvae in the tested group II is presented in Table 3.

The results have indicated that the coordination compound tris[*N*-(prop-2-en-1-yl)hydrazinecarbothioamide]cobalt(III) chloride increased the total antioxidant status in the hemolymph of bees by up to 5.4 times, and in larvae by up to 7.8 times. The IC_50_ values have been recorded at 2.5 mg/mL for bee hemolymph and 1.3 mg/mL for larval hemolymph, compared to control values of 13.6 mg/mL and 10.0 mg/mL, respectively. These results highlight the potential of the coordination compound **[Co(Tsc)_3_]Cl**_3_ as an effective stimulator of the TAS in *Apis mellifera*, contributing to increased resilience of bee colonies against oxidative stress. Due to its antioxidant properties, the coordination compound **[Co(Tsc)_3_]Cl_3_** is of considerable interest for maintaining bee health and productivity.

**Figure 5:**
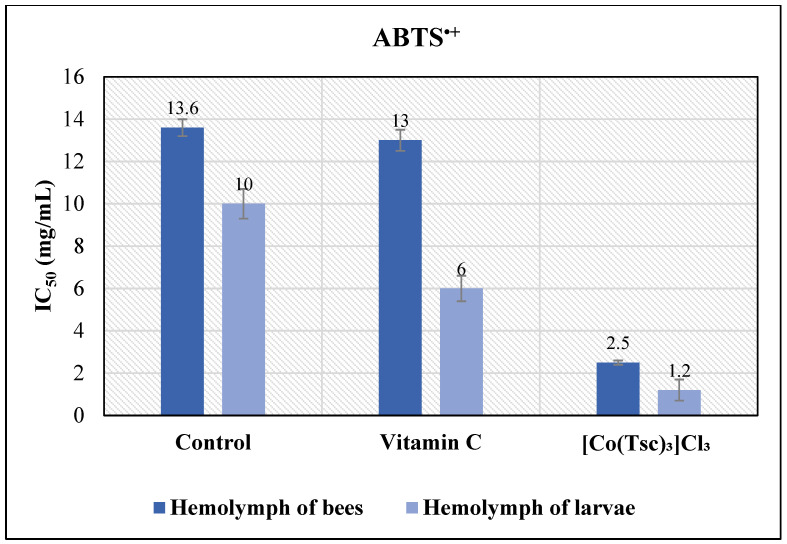
Influence of the coordination compound **[Co(Tsc)_3_]Cl_3_** on the antioxidant property of the hemolymph of larvae and bees.

As shown in Figure 5, the stimulatory effect of the tested compound on TAS exceeded that of vitamin C by up to 5.2 times in bees and up to 4.8 times in larvae.

These findings suggest that increasing TAS levels of bees could enhance their immune systems, highlighting the potential of tris[*N*-(prop-2-en-1-yl)hydrazinecarbothioamide]cobalt(III) chloride as a beneficial additive in apicultural health practices.

The coordination compound **[Co(Tsc)_3_]Cl_3_** effectively neutralizes free radicals, reduces oxidative stress, and helps to protect cellular structures and biomolecules in bees. As a result, it contributes to improved overall health and resilience of bee colonies, especially during periods of environmental stress, such as pollen and nectar scarcity in early spring. This enhancement of antioxidant protection may lead to increased vitality and longevity of the bees, supporting their role as essential pollinators in the ecosystem.

### 3.2. Acaricidal Activity

In recent years, protecting bee colonies from the parasitic mite *Varroa destructor* has become one of the most pressing challenges in beekeeping. According to literature sources, studies have indicated that TAS levels of bees is directly influenced by the degree of infestation by *Varroa destructor.* Infected bees exhibit reduced activity of key enzymes in the antioxidant system, leading to accelerated aging and a shortened lifespan. Particularly severe damage is associated with certain segments of the fat body, highlighting the urgent need to develop protective strategies against parasitic attacks, which is crucial in today’s world [57,58,59].

This external parasitic mite feeds on and reproduces within *Apis mellifera*, causing significant losses in colonies and reducing their health and productivity. Despite the development of new control methods, chemical compounds with high efficacy and low toxicity remain important [60,61,62,63,64,65,66,67], but existing treatments often exhibit insufficient activity. If left uncontrolled, *Varroa destructor* can seriously weaken or destroy most bee colonies [53].

Therefore, our goal is to expand the arsenal of coordination compounds (chemical compounds consisting of a central metal atom or ion bonded to ligands via coordinate covalent bonds, where ligands donate electron pairs, forming a distinct species with unique properties) that can be used as acaricides for the preventive and therapeutic control of *Varroa destructor* infestation in *Apis mellifera* colonies. The article includes an initial screening of the acaricidal properties of a novel coordination compound **[Co(Tsc)_3_]Cl_3_**.

**Figure 6:**
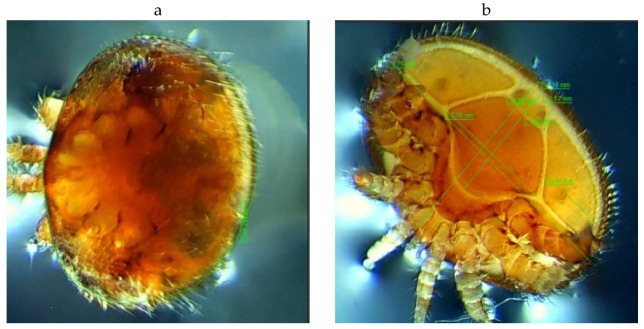
*Varroa destructor* adult female: (**a**) dorsal view, (**b**) ventral view.

The transmission of mites between colonies is facilitated by their morphological and physiological characteristics, which allow them to quickly move from bee to bee. *Varroa destructor* has been identified in apiaries in the Republic of Moldova (Figure 6).

The mite population in the colony grows exponentially, and the greatest damage to the brood occurs in spring when young bees are actively preparing for honey collection. In 2025, the brood infestation level by Varroa in the *Apis mellifera* colonies used in our experiment was notably high, reaching 26.4% in the control group (Table 4). During our trials, up to three mites at various developmental stages were found on larvae. Fewer mites were detected on the bees themselves. These data highlight a critical period when Varroa, being the most dangerous to colony health, requires urgent control measures to limit the rapid increase in population and prevent significant damage to the brood.

For species identification of *V. destructor* mites, we produced morphometry of mites, collected from larvae of *Apis mellifera*. As a result of morphometry, the biometric parameters of indigo and *V. destructor* nymphs were calculated. The average sizes of grayish-white males are L = 0.91 ± 0.15 mm and D = 1.11 ± 0.38 mm. The brown body of the female is strongly sclerotized, flattened in the dorsoventral direction, transversely oval in shape, slightly convex from the side of the dorsal part, and has dimensions L = 1.15 ± 0.05 mm and D = 1.74 ± 0.05 mm. Incompletely sclerotized tritonymphs have dimensions L = 1.11 ± 0.06 mm and D = 1.55 ± 0.12 mm. These parameters correspond to the species identification keys described in De-Guzman, Fernandez, and Coineau (2007) [68].

As part of the biological study on the acaricidal properties of the coordination compound **[Co(Tsc)_3_]Cl_3_** on Apis mellifera colonies, the bees were fed with a syrup composed of 50% sugar and 50% water containing **[Co(Tsc)_3_]Cl_3_** at concentrations of 0.2 mg/L and 0.6 mg/L. The feeding was conducted during the early spring period. Experiments were performed simultaneously on three groups, each comprising 10 hives. Group I served as the control, with bees receiving plain sugar syrup. Groups II and III were experimental, with the bees fed syrup fortified with **[Co(Tsc)_3_]Cl_3_** at concentrations of 0.2 mg/L and 0.6 mg/L, corresponding to doses of 2 mg and 6 mg per hive, respectively. The syrup was administered every two days over a two-week period. The level of Varroa destructor infestation was assessed using a non-destructive diagnostic method based on powdered sugar application. The results of the effect of **[Co(Tsc)_3_]Cl_3_** on mite infestation levels are presented in Table 4.

The data presented in this table reflect the Varroa infestation levels in bees (%) after the treatment period. To accurately assess the acaricidal efficacy of the tested compound, the infestation levels in each hive were compared before and after treatment, accounting for initial differences across colonies, ensuring that the reduction in infestation is evaluated based on within-hive changes rather than initial infestation levels alone. During the two-week experimental period, all colonies were fed with sugar syrup. The control groups received only sugar syrup, while the experimental groups were administered sugar syrup containing the coordination compound **[Co(Tsc)_3_]Cl_3_** at concentrations of 0.2 and 0.6 mg/L. At the start of the experiment, the number of Varroa mites was higher in the hives receiving the coordination compound. After one week, the number of fallen Varroa mites per hive consistently decreased in the treatment groups compared to the control, indicating a lower Varroa load in colonies fed with sugar syrup supplemented with the coordination compound. The level of bee infestation in the control group was 26.4%. After treatment with **[Co(Tsc)_3_]Cl_3_** (0.2 mg/L), the infestation level decreased to 2.7%, and at a concentration of 0.6 mg/L—to 0.4%.

To evaluate the effectiveness of the tested coordination compound **[Co(Tsc)_3_]Cl_3_,** the percentage reduction in *Varroa destructor* infestation levels in *Apis mellifera* colonies was calculated comparative to the control groups. For a comparative characterization of the acaricidal properties of the substances, the median lethal concentration (LC_50_), at which 50% of *Varroa destructor* mites are killed, was determined. The results are presented in Table 5.

Our studies have demonstrated a significant effect: at a concentration of 0.2 mg/L, the coordination compound **[Co(Tsc)_3_]Cl_3_** has reduced the infestation level by 89.8%, and with an increased dose to 0.6 mg/L, the reduction reached 98.5%. Additionally, the LC_50_ of the coordination compound **[Co(Tsc)_3_]Cl_3_** complex was determined to be only 0.2 µmol/L, indicating high acaricidal activity at very low concentration.

Literature sources indicate that CH_3_COOLi (lithium acetate(LiOAc)) possesses acaricidal activity against *Varroa destructor* infestations in honeybee colonies [40]. Consequently, lithium acetate has been identified and established as a acaricidal agent for the control of *Varroa destructor* mites. It has been demonstrated that a concentration of 4 mM lithium acetate achieves complete mite eradication (100% mortality), with a median lethal concentration (LC_50_) of approximately 2.7 µM, while exerting significant adverse effects on bee viability. At a concentration of 0.2 mg/L, **[Co(TSC)_3_]Cl_3_** demonstrates a significantly greater reduction in Varroa destructor infestation, reaching 59.4%, in comparison to LiOAc [40]. The median lethal concentration (LC_50_) of **[Co(TSC)_3_]Cl_3_** for *Varroa destructor* is 13.5 times lower than that of LiOAc, indicating superior acaricidal potency. These results demonstrate the promising potential of the coordination compound **[Co(Tsc)_3_]Cl_3_** as an effective and safe agent for controlling *Varroa destructor* in beekeeping.

The results indicate that compound **[Co(Tsc)_3_]Cl_3_** is a promising, safe, and effective means of controlling *Varroa destructor* in bee colonies. Field studies in apiaries have confirmed its high acaricidal activity under real-world conditions. It has been established that the coordination compound **[Co(Tsc)_3_]Cl_3_** stimulates the overall antioxidant status of the hemolymph of honeybees (*Apis mellifera*) and their larvae, thereby enhancing their resistance to diseases. These findings suggest that the coordination compound **[Co(Tsc)_3_]Cl_3_** can be used as a safe and effective agent for combating *Varroa destructor*, improving the overall health of bees, and increasing their productivity. This approach can significantly reduce parasite populations, improve colony health, and substantially decrease economic losses in beekeeping. However, it is crucial to underscore that these promising results should be validated across different contexts. To fully establish the efficacy and safety of the coordination compound **[Co(Tsc)_3_]Cl_3_**, further studies conducted by independent research groups are essential. These studies should include various honeybee subspecies and ecotypes, as well as colonies situated in diverse climatic regions, to evaluate the broad applicability of this compound. Additionally, future research should employ a wide range of assessment methodologies to accurately measure the acaricidal effectiveness of Varoacid. This includes, but is not limited to, field monitoring of mite populations, biochemical assays, microscopic analyses, and long-term colony health evaluations. Such comprehensive validation is necessary to ensure that the promising outcomes observed in this study can be reliably replicated and applied across different beekeeping environments. Only with rigorous, multi-faceted testing can we confirm the potential of the coordination compound **[Co(Tsc)_3_]Cl_3_** as a safe, effective, and sustainable solution for Varroa control, ultimately contributing to healthier, more productive bee colonies

## 4. Conclusions

The cobalt(III) complex has been synthesized via the reaction of cobalt(II) chloride hexahydrate with *N*-(prop-2-en-1-yl)hydrazinecarbothioamide in ethanol. The antioxidant activity of the complex was evaluated using ABTS^•+^ radical scavenging assay, which demonstrated a significant free radical scavenging capacity, with an IC_50_ value of 7.3 µmol/L, while toxicity assessment with *Daphnia magna* showed low toxicity, with an LC_50_ of 56.3 µmol/L.

Experimental results demonstrated that the coordination compound tris[*N*-(prop-2-en-1-yl)hydrazinecarbothioamide]cobalt(III) chloride increased the total antioxidant status (TAS) in the hemolymph of bees and larvae by up to 5 and 8 times, respectively. The IC_50_ values for antioxidant activity of hemolymph are 2.5 mg/mL for bee and 1.3 mg/mL for larval, significantly lower than control values of 13.6 mg/mL and 10.0 mg/mL, respectively. The stimulatory effect of the tested compound on TAS surpassed that of vitamin C by up to five times.

These findings suggest that boosting TAS levels in bees could enhance their immune response, highlighting the potential of tris[*N*-(prop-2-en-1-yl)hydrazinecarbothioamide]cobalt(III) chloride as a beneficial additive in apicultural practices. Moreover, our studies confirmed that this coordination compound acts as an antioxidant, thereby improving the antioxidative capacity of bee hemolymph and enhancing overall colony resilience.

This finding is of significant scientific relevance as the coordination compound **[Co(Tsc)_3_]Cl_3_** shows a strong acaricidal property, effectively inhibiting *Varroa destructor* with an LC_50_ of 0.2 µmol/L. This highlights the importance of developing new compounds that simultaneously enhance antioxidant defenses and serve as effective acaricides. Such substances are essential for protecting bees from parasitic infections and environmental stressors, meeting the current demand for sustainable and safe beekeeping solutions.

## 5. Patent

Tris[*N*-(prop-2-en-1-yl)hydrazinecarbothioamide]-cobalt(III) chloride as a stimulator of the total antioxidant activity status of hemolymph of *Apis mellifera* bees and their larvae. GARBUZ Olga, MD; GRAUR Vasilii, MD; ŢAPCOV Victor, MD; TODERAŞ Ion, MD; GULEA Aurelian, MD. MD 4905 C1 2025.05.31.

## Figures and Tables

**Table 1:** ABTS^•+^ scavenging activity of the complex **[Co(Tsc)_3_]Cl_3_**, Trolox, vitamin C.

Compound	ABTS^•+^ IC_50_ (µmol/L)
**Trolox**	33.3 ± 0.3
**Vitamin C**	14.0 ± 0.3
**[Co(Tsc)_3_]Cl_3_**	7.3 ± 0.3

**Table 2:** TAS of hemolymph of worker bees and their larvae of the control group I.

Hemolymph of Bees C (mg/mL)	ABTS^•+^ Inh. (%)	ABTS^•+^ IC_50_ (mg/mL)	DPPH^•^ Inh. (%)	DPPH^•^ IC_50_ (mg/mL)
100.0	81.43 ± 2.02	13.56 ± 0.37	90.18 ± 1.09	13.85 ± 0.19
10.0	42.56 ± 2.27		41.37 ± 1.26	
1.0	16.49 ± 0.42		5.42 ± 0.25	
0.1	4.17 ± 0.51		0.71 ± 0.51	
**Hemolymph of larvae** **C (** **mg/mL)**				
50.00	92.74 ± 4.55	9.95 ± 0.70	95.89 ± 1.77	13.44 ± 2.95
5.00	25.36 ± 1.68		9.29 ± 4.88	
0.50	10.06 ± 0.08		6.73 ± 109	
0.05	0.54 ± 0.04		4.23 ± 1.77	

**Table 3:** TAS of hemolymph of worker bees in the tested group II.

Hemolymph of Bees C (mg/mL)	ABTS^•+^ Inh. (%)	ABTS^•+^ IC_50_ (mg/mL)	DPPH^•^ Inh. (%)	DPPH^•^ IC_50_ (mg/mL)
100.0	94.76 ± 0.17	2.48 ± 0.22	74.94 ± 1.26	7.08 ± 0.34
10.0	92.08 ± 1.25		55.00 ± 0.51	
1.0	17.14 ± 0.01		32.02 ± 0.34	
0.1	3.69 ± 0.17		7.80 ± 0.08	
**Hemolymph of larvae** **C (** **mg/mL)**				
50.00	94.38 ± 0.04	1.26 ± 0.12	95.65 ± 1.77	6.70 ± 0.12
5.00	89.44 ± 1.68		38.81 ± 0.15	
0.50	19.69 ± 0.09		11.67 ± 0.05	
0.05	5.25 ± 0.35		3.69 ± 1.25	

**Table 4:** Varroa infestation levels in bees (%) in the control groups and in the groups with the addition of the coordination compound **[Co(Tsc)_3_]Cl_3_** at concentrations of 0.2 and 0.6 mg/L after the treatment period.

Number of Colonies	Varroa Infestation Levels/100 Bees		
	Control Groups	[Co(Tsc)_3_]Cl_3_ (0.2 mg/L)	[Co(Tsc)_3_]Cl_3_ (0.6 mg/L)
**1**	32	4	0
**2**	25	3	0
**3**	37	4	1
**4**	26	2	0
**5**	28	1	1
**6**	24	5	1
**7**	30	1	0
**8**	20	2	0
**9**	24	2	1
**10**	18	3	0
**M**	**26.4**	**2.7**	**0.4**

**Table 5:** The percentage reduction in *Varroa destructor* infestation levels in *Apis mellifera* colonies and LC_50_, μM of **[Co(Tsc)_3_]Cl_3_**.

Compound	Percentage Reduction in the Infestation Level of *Apis mellifera* Bees with *Varroa destructor* Mites		LC_50_ (µmol/L)
	(0.2 mg/L)	(0.6 mg/L)	
**[Co(Tsc)_3_]Cl_3_**	89.8	98.5	0.2

## Data Availability

The original contributions presented in this study are included in the article. Further inquiries can be directed to the corresponding authors.

## Abbreviations

The following abbreviations are used in this manuscript:

[Co(Tsc)_3_]Cl_3_	tris[*N*-(prop-2-en-1-yl)hydrazinecarbothioamide]-cobalt(III) chloride
ABTS^•+^	2,2′-azino-bis(3-ethylbenzothiazoline-6-sulphonic acid)
DPPH^•^	1,1-diphenyl-2-picrylhydrazyl
TAS	Total antioxidant status
AOA	Antioxidant activity
Tsc	4-allylthiosemicarbazide
I, %	Percent of inhibition
ROS	Reactive oxygen species
SD	Standard deviation
IC_50_	Half-maximal inhibitory concentration
LC_50_	Median lethal concentration
